# IL-17A polymorphism (rs2275913) and levels are associated with preeclampsia pathogenesis in Chinese patients

**DOI:** 10.1186/s12920-020-00840-8

**Published:** 2021-01-06

**Authors:** Xiao Lang, Wei Liu, Yanyan Hou, Wenxia Zhao, Xingyu Yang, Lan Chen, Qi Yan, Weiwei Cheng

**Affiliations:** 1 The International Peace Maternity and Child Health Hospital, School of Medicine, Shanghai Jiao Tong University, 910 Hengshan Road, Shanghai, 200030 China; 2 Shanghai Municipal Key Clinical Specialty, Shanghai, 200030 China; 3 Department of Obstetrics and Gynecology, Shanghai Fourth People’s Hospital, Tongji University, Shanghai, 200030 China; 4 Shanghai Key Laboratory of Embryo Original Diseases, Shanghai, 200030 China

**Keywords:** Preeclampsia, IL-17A, IL-23A, Gene polymorphism, Serum levels

## Abstract

**Background:**

Preeclampsia (PE) is a pregnancy-related condition that affects both the infant and the mother. Although the role of various inflammatory molecules in PE has been demonstrated, the importance of pro-inflammatory molecules such as IL-17A, IL-23 is not well understood. In the present investigation, a potential association of common genetic variants in the IL-17A and IL-23A genes with PE was investigated.

**Methods:**

115 PE clinically diagnosed patients who registered to the International Peace Maternity and Child Health Hospital were enrolled in this research. One hundred two pregnant women and 147 healthy Chinese women were also included. ELISA was used to measure IL-17A and IL-23 serum levels in all enrolled subjects. Common genetic polymorphisms in *IL-17A* (rs 2,275,913, rs1974226, and rs1974226), *IL-23A* (rs11171806), and *IL-12B* (rs3212227) were genotyped using the PCR-RFLP or TaqMan probe-based method.

**Results:**

Elevated serum IL-17A levels were found in PE patients compared to pregnant (*P* < 0.0001) and healthy women (*P* < 0.0001). However, IL-23 levels were comparable across various clinical groups. In addition, heterozygous (GA) and minor allele (A) for IL-17A (rs2275913) and IL-23A (rs11171806) were more prevalent in PE patients compared to pregnant women indicating an important role in the predisposition to PE growth. Interestingly, IL-17A (r 2,275,913) mutants were associated with elevated IL-17A levels relative to wild type (GG).

**Conclusions:**

IL-17A (rs2275913) variants are associated with higher serum levels of cytokine, and predisposed PE development.

## Background

Preeclampsia (PE) is a complication associated with pregnancy and is characterised by high blood pressure and dysfunction of different organ systems. PE syndrome has a deleterious effect on both the mother and the developing foetus. Although the exact cause and pathogenesis of this disease are not known, it is suspected that the disease is guided by a variety of factors caused by placental pressure-induced trophoblasts, which promote overwhelming maternal inflammatory response [1]. It is estimated that about 10 million people are developing PE per year worldwide [2]. In addition, approximately 76,000 pregnant women die each year as a result of PE and approximately 500,000 children die annually as a result of PE [3]. Epidemiological studies of PE in the Chinese population are very limited: a retrospective analysis in three separate hospitals in China found around 2.35% PE in a total of 67,746 pregnant women and PE was most prevalent in nulliparity subjects (81.5%) [4]. There is a wide repertoire of immunological modulators and signalling pathways that lead to the initiation and progression of PE. Previous evidences indicate that cytokines play a critical role in controlling various stages of pregnancy among different immune molecules [5, 6]. Th1: Th2 dichotomy during pregnancy indicated that Th2 mediated immunity is involved in the maintenance of normal occurrences during pregnancy, while Th1 form of immune response is associated with pregnancy-related problems such as miscarriage [7], premature delivery [8], rupture of foetal membranes prior to the start of labour pain [9]. Previous studies have suggested a difference in preeclamptic placenta levels of cytokines, leading to complicated conditions such as delayed intrauterine development and preterm delivery [10]. Cytokine IL-17 was derived from Th17 lymphocytes recently discovered as a subset of CD4 + T lymphocytes [11]. Several studies indicated a higher percentage of the population of Th17 cells in complicated pregnancy cases, such as abortion, preterm birth or PE [12, 13, 14].

Interlukin-23 (IL-23) is a proinflammatory cytokine that is responsible for Th-17 cell discrimination, spread, and survival [15]. In the pathogenesis of PE, upregulation of the Th-17 cell-mediated immune response has been demonstrated [16]. IL-23 is a cytokine heterodimer consisting of subunits IL-12B and IL-23A. There have been earlier reports of differential levels of IL-23 in preeclamptic patients [12]. Based on the importance of IL-23 in controlling inflammation, we hypothesised that IL-23 cytokine could be linked to the clinical condition of PE patients in a Chinese cohort,.

Functional single nucleotide polymorphisms (SNPs) have been associated with the variance of serum cytokine levels in subjects. Although several SNPs are reported in the IL-17A gene, common polymorphisms like rs2275913 (−197G > A), rs1974226 (3’UTR C > T), and rs3748067 (1249C > T) are widely investigated on genetic association studies. Similarly, variants of IL-23A (rs11171806: A > G, exon 106Ser > Ser) and IL-12B (rs3212227 A > C 3’UTR) also investigated in various reports and their association with susceptibility to a wide range of diseases has already been established. Investigations to decipher genetic association of IL-17A, IL-23, and IL-12B common variants with PE is limited. Earlier reports failed to demonstrate possible association of IL-17A polymorphism (rs2275913) with susceptibility to PE in iraninan and Chinese women [17, 18]. However, other functional SNPs in the IL-17A gene has not been explored. Further, IL-12B (rs3212227) polymorphism was also not linked with PE predisposition in Han Chinese [19]. To best of our knowledge, association of IL-23A genetic variants with PE predisposition has not been studied.

Simultaneous investigation into the relationship between IL-17A, IL-23A and IL12B common genetic polymorphisms with predisposition to PE and their respective serum levels with pathogenesis of PE is missing in the Chinese population. In this study, a hospital-based case control investigation was conducted to decipher the combination of IL-17A, IL-23A and IL12B genetic variants and their functional significance in PE pathophysiology.

## Methods

### Study subjects

The present study was performed between a period from March 2017 to December 2019 at the International Peace Maternity and Child Health Hospital. The study protocol was approved by the Institutional Review Board of Shanghai Jiao Tong University and the informed signed consent was obtained from all participants. The present investigation was a hospital based case control study. The first category included 120 patients with PE. The primary inclusion criteria for these patients was third-trimester pregnancy complicated with PE, blood pressure > 140/90 with proteinuria > 300 mg in 24 h (According to ACOG) [20]. The second category included 120 women in third-trimester pregnancy without PE. The third category included 150 healthy non-pregnant women and was used as controls. Patients with microvascular complications, co-existing autoimmune, chronic/acute inflammatory diseases, multiple gestations, diabetes mellitus, sickle cell disease, and HIV infection were excluded from the present investigation. Clinical characteristics data was gathered from hospital records.

### Collection of serum

Three milliliters of blood samples (without anticoagulant) collected intravenously from pregnant women and non-pregnant controls before starting therapy. The serum was separated from each sample by centrifuging blood at 950 g for 5 min. The supernatant was collected and retained at -80 °C for future quantification of cytokines.

### Cytokines (IL-17A and IL-23) quantification

Serum levels IL-17A or IL-23 were quantified by enzyme-linked immunosorbent assays (ELISA) using the pre-designed kit as per the manufacturer’s instructions (R&D Systems, Inc., USA) in all subjects enrolled for the present investigation.

### Genomic DNA isolation

For genomic DNA isolation, 200ul of whole blood was used. SIGMA mini Genomic DNA extraction kit was used for isolation of whole genomic DNA.

### Genotyping of IL-17A, IL-12B and IL-23A polymorphisms

A polymerase chain reaction followed by restriction fragment length polymorphism (PCR-RFLP) technique was employed for the genotyping of IL-12B (rs3212227), as described by an earlier report [21]. Briefly, two primers (forward: GATATCTTTGCTGTATTTGTATAGTT and reverse: AATATTTAAATAGCATGAAGGC) were used for amplification of a 118 bp gene fragment flanking the polymorphic site. The thermal cycler conditions were as follows: early denaturation at 95 °C for 5 min followed by 35 cycles of 95 °C for 40 s, 55 °C for 35 s, and at 72 °C for 25 s. The final extension reaction was performed at 72 °C for 10 min. The amplicon was digested with TaqI restriction enzyme and fragments were analysed for rs3212227 polymorphism. Amplicon with mutant allele has a restriction site for TaqI, thus produces two fragments (92 bp + 26 bp); on the other hand, for wildtype allele, the amplicon remained undigested (118 bp). Similarly, the PCR-RFLP technique was also used for genotyping of IL-17A (rs2275913, rs1974226, and rs3748067) polymorphisms, as described in an earlier report [22]. Following primers were used for amplification of IL-17A gene bordering SNPs sites and yield different amplicons (rs2275913: forward- GCTCAGCTTCTAACAAGTAAG, reverse- AAGAGCATCGCACGTTAGTG, amplicon size-338 bp; rs1974226: forward- AAAGGAGCTGATGGGGCAGTA, reverse- GGTCTTTCAAGAAGCAGGGAG, amplicon size-211; rs3748067: forward- GGGCTGAACTTTTCTCATACTTAGA, reverse- GAGACATTGTCTTCAGACTACAATG, amplicon size-212 bp). The annealing temperature for genotyping of IL-17A polymorphisms was fixed at 58 °C, and other conditions were like those of IL-12B. Different restriction enzymes were used (rs2275913: EarI, rs1974226: RsaI and rs3748067: EcoRI) for digestion of the amplicon and based on differential digested DNA fragments, genotypes of subjects were determined as follows (rs2275913: A = 259 + 79 bp, G = 338 bp; rs1974226: A = 221 bp, G = 191 + 20 bp and rs3748067:G = 212 bp, A = 198 + 24 bp). As described earlier [23], the IL-23A (rs11171806) gene polymorphism was genotyped by the TaqMan PCR assay by using a TaqMan probe. In brief, predesigned SNP genotyping assays kit were procured from Thermo Fisher Scientific (rs11171806: C__25985467_10, VIC/FAM- TTTTTTATGAGAAGCTGCTAGGATC[A/G]GATATTTTCACAGGGGAGCCTTCTC) and the typing was performed in Applied Biosystems Realtime PCR system (7900HT) as directed by the manufacturer.

### Statistical analysis

Graphpad prism v8.2 was employed for all statistical analyses. Serum levels of IL-17A and IL-23 in different clinical groups were compared by one-way analysis of variance (ANOVA) and the mean cytokines levels of all groups were compared with those of every other clinical categories or genotypes by Tukey’s post-test. Genotype and allele frequencies were calculated by manual counting. Genotypes distribution of all studied SNPs were tested for Hardy Weinberg equilibrium with in house developed Microsoft excel file. Distribution of genotypes and alleles were compared with Fisher exact test in different clinical categories, odds ratio and 95% confidence interval was calculated. A *P* value of less than 0.05 was taken as significant.

## Results

### Baseline characteristics of patients and controls

A total of 120 cases of PE and 120 pregnant women were included in the study. Both plasma cytokines and genetic polymorphisms was successfully analysd in 115 PE patients and 102 pregnant women. Out of 150 healthy Chinese women included in the present investigation, IL-17, IL-23 and IL-12B polymorphisms and plasma cytokines levels were efficiently quantified in 147 subjects. Thus based on availability of data for both genotypes and levels of cytokines, a total of 364 females were considered in the present study comprising 115 PE patients, 102 pregnant women, and 147 non-pregnant women. Different baseline characteristics were compared among clinical categories (Table 1). A significant difference was observed in different parameters while comparing PE cases and pregnant women, such as duration of gestation (days), body mass index (Kg/m2), vaginal delivery (%), caesarian section (%), fetal birth weight (gram), systolic/diastolic blood pressure (mmHg), WBC count (× 10^9^/ L), levels of urea and uric acid (mg/dL). However, percentage of pre-eclamptic patients with primiparas were not significantly altered in comparison to healthy pregnant women.

**Table 1:** Baseline characteristics of study subjects

Parameters	Subjects with PE (*n* = 115)	Pregnant women (*n* = 102)	Non-pregnant women (*n* = 147)
Age (years)	31 ± 5	33 ± 6	29 ± 4
Primiparas (%)	59.3	54.9	NA
Duration of gestation (days)	249 ± 14*	269 ± 19	NA
BMI at blood draw (Kg/m2)	30.1 ± 5.2*#	26.2 ± 4.1#	21.1 ± 3.8
Vaginal delivery (%)	12.3*	56.6	NA
Caesarian section (%)	87.7*	43.4	NA
Fetal birth weight (grams)	2651*	3216	NA
Systolic blood pressure (mmHg)	149 ± 13*#	109 ± 09	113 ± 11
Diastolic blood pressure (mmHg)	89 ± 11*#	75 ± 09	80 ± 10
White blood cell (× 10^9^/L)	10.1 ± 3.5*#	9.6 ± 3.2#	8.8 ± 2.9
Uric acid (mg/dL)	7.1 ± 0.9*#	4.2 ± 1.3	3.9 ± 1.9
Urea (mg/dL)	24.09 ± 13.6*#	18.6 ± 3.9	17.6 ± 4.4

Data are presented as either mean ± S.D. or in percentage (%)

*NA* Not applicable

* *P* < 0.05- Subjects with PE versus healthy pregnant women; # *P* < 0.05 - Subjects with PE versus healthy non pregnant women

### PE patients displayed higher serum IL-17A compared to controls

**Fig. 1:**
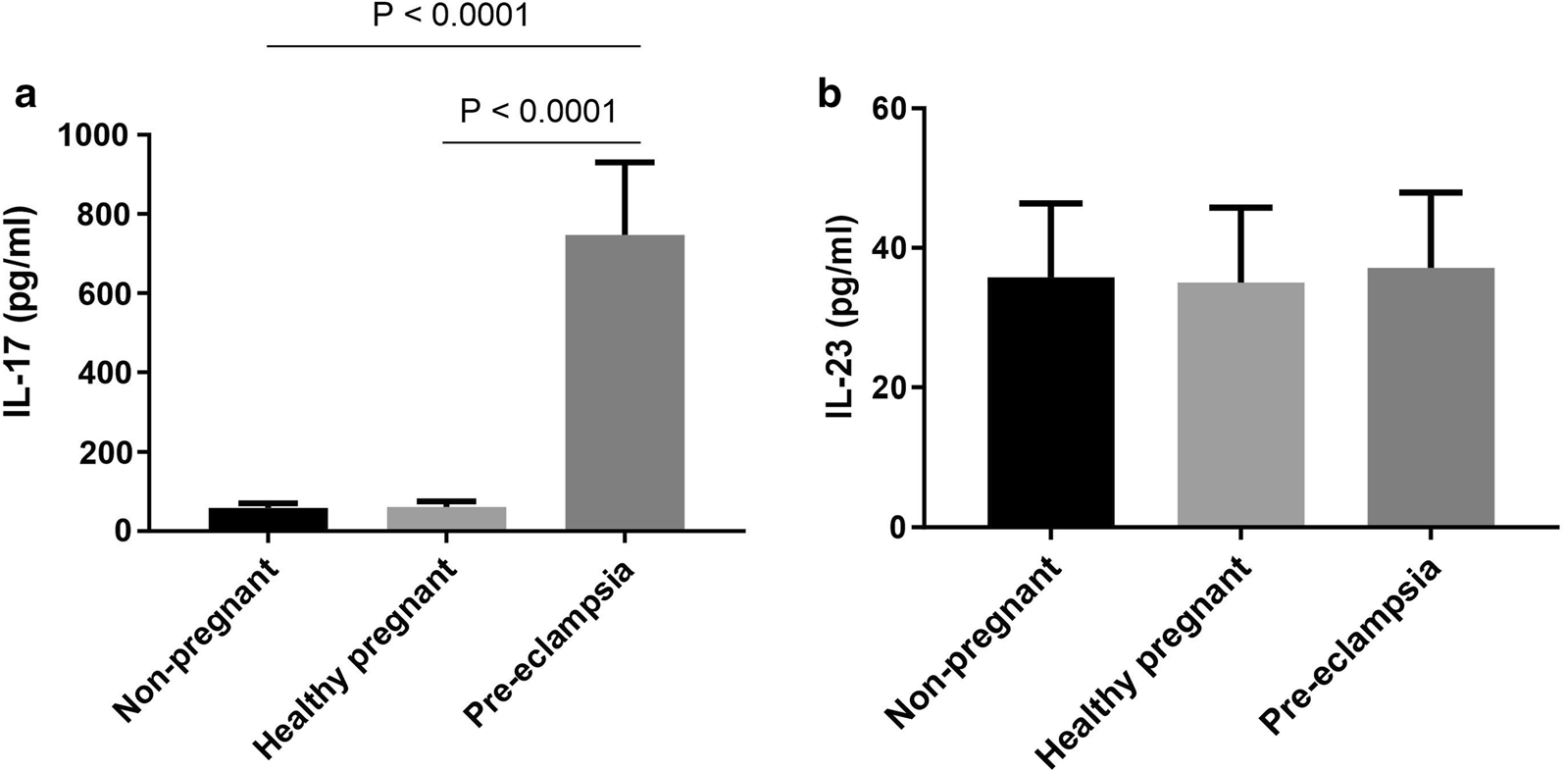
Serum cytokines levels in different categories of enrolled subjects. Serum level IL-17 (**a**) and IL-23 (**b**) in subjects with PE (*n* = 115), healthy pregnant women (*n* = 102) and healthy non pregnant controls (*n* = 147). Data represent mean pg/ml ± SE and were analyzed with one way ANOVA for comparison. *P* < 0.05 was considered statistically significant

The serum levels of IL-17A and IL-23 were quantified by ELISA. As shown in Fig. 1a, the mean level of IL-17A was 59.1 ± 0.93 pg/ml in healthy women without pregnancy, whereas healthy pregnant women and subjects with PE had 61.13 ± 1.43 pg/ml and 746.7 ± 17.16 pg/ml of IL-17A level, respectively. Although a comparable level in IL-17A was observed between healthy pregnant and non-pregnant women, subjects with PE demonstrated a noticeably higher cytokine level as compared to two other study groups, suggesting an essential role of this molecule in promoting pathogenesis during PE. Further, to assesses the importance of IL-23 in regulating the pathologic condition of PE, the titer of the cytokine was measured in sera and the results are shown in Fig. 1b. The results showed a relatively similar level of this cytokine in all the three groups.

### Distribution of IL-17A, IL-23A and IL-12B polymorphisms in healthy non-pregnant women

The genotype and allele frequency of IL-17A (rs2275913, rs1974226, and rs3748067), IL-23A (rs11171806) and IL-12B (rs3212227) polymorphisms in healthy female and the distribution of genotypes for all SNPs are in Hardy-Weinberg equilibrium (rs2275913: *X*^2^ = 2.58, *P* = 0.10; rs1974226: *X*^2^ = 2.83, *P* = 0.08; rs3748067: *X*^2^ = 0.17, *P* = 0.67; rs11171806: *X*^2^ = 1.93, *P* = 0.16; rs3212227: *X*^2^ = 0.01, *P* = 0.90) (Table 2).

**Table 2:** Distribution of IL-23A, IL-12B and IL-17A gene polymorphisms in healthy controls, pregnant woman and preeclampsia patients

Genotype/Allele	HW (*n* = 147)	PW (*n* = 102)	PE (*n* = 115)	PW vs PE (*P* value, OR, 95% CI)
IL-23A (rs11171806)				
GG	135 (92)	95 (93)	92 (80)	1, ref
GA	11 (7)	7 (7)	22 (19)	**0.008, 3.24, 1.31 to 8.53**
AA	1 (1)	0	1 (1)	–
G	281 (96)	197 (97)	206 (90)	1, ref
A	13 (4)	7 (3)	24 (10)	**0.004, 3.27, 1.44 to 8.18**
IL-12B (rs3212227)				
AA	125 (85)	89 (87)	93 (81)	1, ref
AC	21 (14)	12 (12)	20 (17)	0.25, 1.59, 0.75 to 3.47
CC	1 (1)	1 (1)	2 (2)	1, 1.91, 0.21 to 28.01
A	271 (92)	190 (93)	206 (90)	1, ref
C	23 (8)	14 (7)	24 (10)	
IL-17A (rs2275913)				
GG	54 (37)	39 (38)	24 (21)	1, ref
GA	62 (42)	44 (43)	65 (56)	**0.007, 2.40, 1.29 to 4.47**
AA	31 (21)	19 (19)	26 (23)	1, 0.96, 0.47 to 1.96
G	170 (58)	122 (60)	113 (49)	1, ref
A	124 (42)	82 (40)	117 (51)	**0.02, 1.54, 1.05 to 2.25**
IL-17A (rs1974226)				
CC	129 (88)	87 (85)	102 (89)	1, ref
CT	16 (11)	13 (13)	13 (11)	0.83, 0.85, 0.37 to 1.94
TT	2 (1)	2 (2)	0	–
C	274 (93)	187 (92)	217 (94)	1, ref
T	20 (7)	17 (8)	13 (6)	0.34, 0.65, 0.32 to 1.36
IL-17A (rs3748067)				
CC	119 (81)	82 (80)	91 (79)	1, ref
CT	26 (18)	20 (20)	24 (21)	0.86, 1.08, 0.56 to 2.08
TT	2 (1)	0	0	–
C	264 (90)	184 (90)	206 (90)	1, ref
T	30 (10)	20 (10)	24 (10)	0.87, 1.07, 0.58 to 2.04

Data are presented in number (%)

*HW* Healthy non-pregnant woman, *PW* Pregnant woman, *PE* Preeclampsia patients, *OR* Odds ratio, *CI* Confidence interval

### IL-17A (rs2275913) and IL-23A (rs11171806) polymorphisms are associated with predisposition to PE

As shown in Table 2. Heterozygous (GA) and minor allele (A) of IL-17A (rs2275913) polymorphism were significantly more prevalent in PE patients compared to the pregnant women (GA: *P* = 0.007, OR = 2.40; A: *P* = 0.02, OR = 1.54). No significant genetic association was observed in the distribution of other IL-17A polymorphisms (rs1974226 and rs3748067) and IL-12B (rs3212227) in PE patients in comparison to the pregnant women. Interestingly, when we analyzed association of IL-23A polymorphism (rs11171806) with predisposition to development of PE, a significant link of heterozygous variants and minor allele were noticed with susceptibility to PE development (GA: *P* = 0.008, OR = 3.24, A: *P* = 0.004, OR = 3.27).

### Genotype-phenotype association of IL-17A and IL-23A polymorphisms

**Fig. 2:**
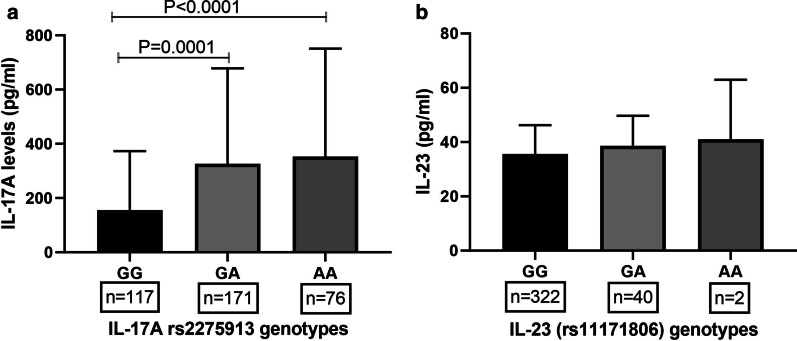
Association of IL-17A (rs2275913) and IL-23 (rs11171806) polymorphism with respective serum levels. Serum levels of IL-17A were quantified by ELISA and rs2275913 polymorphism was typed by PCR-RFLP in a total of 364 subjects comprising of healthy females, pregnant woman, and subject with preeclampsia (**a**). Mean IL-17A levels in the different genotype of rs2275913 polymorphism were compared by ANOVA followed by Tukey’s post-test. No significant association between IL-23A (rs11171806) and levels of IL-23 was observed (**b**). A *P* value of less than 0.05 was taken as significant

AA and GA genotypes of rs2275913 polymorphisms displayed significantly higher serum IL-17A compared to wildtype (GG) (Fig. 2a). Serum IL-17A levels were comparable among different genotypes of rs1974226 and rs3748067 polymorphisms (data not shown). Furthermore, variants of IL-23A (rs11171806) also failed to demonstrate any functional relevance on serum levels of IL-23A (Fig. 2b).

## Discussion

The importance of proinflammatory molecules in PE pathogenesis has been deciphered by several studies. However, the role of IL-17 and IL-23 in PE has not been extensively investigated. In addition, the association of common polymorphisms with PE predisposition and levels of the respective cytokines was never analysed in Chinese. We have genotyped common variants of IL17A, IL-23 and IL-12B and serum cytokine levels in Chinese PE patients and controls in the present study. Current reports shows that IL-17A (rs2275913) polymorphisms are associated with higher serum IL-17A levels and a predisposition to PE.

We observed elevated IL-17A levels in PE patients in comparison to the pregnant ladies and healthy cases. However, earlier reports remained controversial concerning the importance of IL-17A in PE. For example, a study by Jonsson et al. [24] showed no evidence of possible links between IL-17A levels and PE pathologic conditions. On the contrary, our result is corroborated with earlier observations indicating a remarkably higher titer of IL-17A in the sera of pregnant subjects complicated by fetal growth restriction (FGR) and PE as compared to healthy pregnant normotensive women [25]. These results were further strengthened by several other reports mentioning that there was a higher prevalence of Th17 cells in peripheral blood and enhanced expression of RORγt mRNA (transcription factor of Th17 cells) in placentas of pre-eclamptic subjects as compared normal healthy ones [12, 26, 27]. Gaestational period could also be a possible reason for the differential IL-17A levels between PE patients and healthy pregnant women. Earlier investigations in the experimental model have deciphered the essential role of IL-17 in PE pathogenesis. Infusion of IL-17 to normal pregnant rats increased mean arterial pressure, elevated oxidative stress and enhanced Th17 cells [28]. Administration of Rituximab and superoxide dismutase to IL-17 infused rats improved pathogenesis by lowering the number of Th17 cells [28]. In addition, the administration of soluble IL-17 receptor significantly decreased Th17 cells, lowered blood pressure, and improved pathophysiological status of infused IL17 rats [29].

In contrast, we did not observe a possible difference in serum level IL-23 levels among three different studied groups. These results are contradictory to an earlier report [30] where a significantly lower level of this cytokine was demonstrated in pregnant groups (with and without PE) in comparison to healthy non-pregnant subjects. However, in line with our observations, a report by Darmochwal-Kolarz et al. [25] also failed to demonstrate the difference of IL-23 among pregnant subjects with placental insufficiency (fetal growth restriction and PE) and healthy pregnant women. Our result showing a comparable level of IL-23 in subjects with PE and healthy pregnant women matched with the report, as mentioned above. Moreover, a comparable level of this cytokine in both the pregnant groups and healthy non-pregnant subjects indicate a negligible role of IL-23 in the context of pregnancy and its related complication in PE.

Common polymorphisms in the IL-17A gene have been associated with hypertension [31] and various organ dysfunctions [32]. As the primary clinical characteristics of PE are high blood pressure and dysfunction of kidney and liver, we hypothesized that variants in the IL-17A gene would be associated with predisposition to the development of PE. Out of three SNPs investigated in the present study, we observed a significant association of rs2275913 polymorphism with a predisposition to PE: heterozygous and minor allele was more frequent in PE cased when compared to pregnant women and healthy women. In contrast, the previous reports in Brazilian [33], Han Chinese [18], and the Iranian population [17] failed to demonstrate such association. Similarly, other variants of IL-17A polymorphism (rs1974226 and rs374806) were also not associated with susceptibility to PE. Furthermore, IL-23A (rs11171806) variants were also more frequent in PE compared to healthy and pregnant women indicating a susceptible genetic factor for PE development. The possible mechanism of how IL-23A rs11171806 is associated with PE susceptibility is not known. Mutation at 703 nucleic acids (G > A) position leads to a synonymous Ser106Ser, not affecting the three-dimensional structure of the IL-23 protein. Furthermore, in the present study, we also failed to observed differential IL-23 serum levels among different clinical categories. Also, the distribution of IL-12B (rs3212227) variants was comparable in PE patients and other clinical categories, indicating no significant role of IL-12B polymorphism in predisposition to PE development.

In the current report, a strong association of IL-17A (rs2275913) and IL-23A (rs11171806) polymorphism with susceptibility to PE was observed. Also, we noticed elevated IL-17A levels in PE patients in comparison to healthy women and pregnant women, and IL-23 remained comparable. Based on these results, we hypothesized that common polymorphisms in IL-17A and IL-23A would be correlated with serum levels of IL-17A and IL-23, respectively. Serum levels of IL-23 were not associated with different genotypes of IL-23A gene (rs11171806), as the mutation (G > A) lead to no change in amino acids (Ser106Ser). Interestingly, IL-17A (rs2275913) polymorphism was observed to contributing serum levels of IL-17A: homozygous (AA) and heterozygous mutant (GA) displayed higher serum IL-17A compared to GG genotype. In line with the present report, earlier studies [31, 34] have also demonstrated the functional relevance of IL-17A (rs2275913) with plasma or serum levels of IL-17A. Genetic variation at the promoter region of IL-17A gene would possibly enhance the binding of transcription factor and increased production of IL-17A cytokine.

## Conclusions

The current report revealed an important role of IL-17A in the pathogenesis of PE in Chinese patients. Furthermore, heterozygous mutant and minor allele of IL-17A (rs2275913) and IL-23A (rs11171806) polymorphisms predisposed subjects for the development of PE. Interestingly, the current report further re-validated the functional relevance of IL-17A (rs2275913) variants and demonstrated the association of mutants with elevated IL-17A levels. However, further studies, including more significant sample-sized in the different populations, are required to validate the observations of the present study.

## Data Availability

The datasets used and/or analysed during the current study are available from the corresponding author on reasonable request.

## Abbreviations

ELISA: Enzyme-linked immunosorbent assay
IL: Interlukin
